# *Dermkielia cavawata* sp. nov. gen. nov., Isolated From Plantar Eczema

**DOI:** 10.1111/myc.70218

**Published:** 2026-08-24

**Authors:** Jochen Brasch, Yvonne Gräser, Karen Voss, Stephan Weidinger, Andrey Yurkov

**Affiliations:** ^1^ Department of Dermatology University Hospitals of Schleswig‐Holstein Kiel Germany; ^2^ Institute of Microbiology, Infectious Diseases and Immunology, Charité Berlin Germany; ^3^ Leibniz Institute DSMZ German Collection of Microorganisms and Cell Cultures GmbH Braunschweig Germany

**Keywords:** habitat, phenotype, phylogeny, physiology, *Pseudogeomyces*, skin, *Thelebolaceae*

## Abstract

**Background:**

Human skin is naturally exposed to diverse fungi. New taxa, including species with unknown pathogenic potential, continue to be isolated from human skin.

**Objective:**

To characterize a hitherto unknown hyphomycete isolated from human eczema and to assess its habitat and pathogenic abilities.

**Methods:**

A broad panel of culture conditions was used for morphological examination and physiological testing. Nucleotide ribosomal ITS and LSU sequences were analyzed separately by BLASTn to identify the closest relatives, and a combined ITS‐LSU dataset was used for phylogenetic inference.

**Results:**

The isolate grew well on common mycological agars at 26°C and was not inhibited by 0.4 g/L cycloheximide. Colonies were flat, with a beige obverse and sparse aerial mycelium. Intercalary, round to subglobose spores developed within the hyphae, and submerged hyphae in cultures older than 4 weeks transformed into chains of spores consistent with chlamydospores. Neither microconidia, macroconidia, nor ascomata were observed. The isolate showed urease, keratinolytic, proteolytic, lipolytic and cellulolytic activity. It grew at 5°C and tolerated up to 6% NaCl; susceptibility to terbinafine was reduced. Phylogenetically, the isolate formed a distinct lineage branching basally to *Pseudogeomyces*, separated from related taxa by a distance comparable to that between *Geomyces* and *Solomyces*.

**Conclusions:**

*Dermkielia* gen. nov. (IF 905143) is introduced with *Dermkielia cavawata* sp. nov. (IF 905164) as the type species. Related ITS sequences in NCBI GenBank suggest that additional species of *Dermkielia* may be discovered. *Dermkielia cavawata* is presumably geophilic and possibly linked to forest ecosystems, but it possesses traits that may facilitate opportunistic colonization of human keratinized tissue.

## 1 Introduction

Human skin is naturally exposed to a wide diversity of microorganisms, including fungal propagules derived from the environment and the resident microbiota. The resident fungal microbiota is dominated by lipophilic yeasts of the genus *Malassezia*, especially at sebaceous body sites [1]. In addition, Saccharomycotina yeasts may be present as commensals at mucocutaneous junctions and in moist skin areas. They are regarded as opportunists, although equipped with serious pathogenic potential [2]. Overall, the composition of the cutaneous mycobiota varies with anatomical site, host age and environmental factors [3]. It may also include other yeasts and filamentous fungi at lower abundance when local conditions permit. Most fungal species are unable to persist on the skin surface, which is an unfavourably dry and continuously renewing habitat. However, impairment of epidermal barrier function due to trauma, infection and inflammation may allow taxa otherwise associated with soil, plants and other habitats to colonize or infect human skin. In many cases, adherence to the stratum corneum is a pioneering first step in this process [4].

The ability of fungi to survive on human skin is closely linked to the structural and biochemical properties of the outermost epidermal barrier, whose mechanical resilience largely depends on keratin‐rich corneocytes. Accordingly, the production of keratinases is regarded as an important trait in skin‐pathogenic fungi [5, 6]. This relationship is most clearly exemplified by dermatophytes; a group of fungi specialized for growth on skin with pronounced keratinolytic activity that can cause tinea [4]. However, the ability to degrade keratin is also present in many fungi adapted to very different habitats, like soil or plants. Reflecting such distinct ecological preferences, clades and genera were defined that integrate their environmental adaptation (human‐associated, animal‐borne or terricolous) into taxonomic classification. Accordingly, several major human pathogens are now placed in the order *Onygenales* [7, 8]. Beyond this order, many further genera (e.g., *Aspergillus, Fusarium, Cryptococcus, Mucor*, to name but a few) comprise species capable of causing severe and even invasive disease under favouring conditions.

In recent years, a growing number of keratinolytic non‐dermatophytes has been collected from human skin and nails, underlining that the spectrum of fungi capable of exploiting keratinized tissues is broader than previously assumed [9, 10]. Although many of these organisms are probably only transient skin colonizers or opportunists rather than primary pathogens, their isolation from diseased skin highlights the need for closer taxonomic and clinical evaluation. Furthermore, reliable identification of these fungi is important as their distant evolutionary relatedness to most dermatophytes may be associated with different antifungal susceptibility profiles and, consequently, different therapeutic treatments. In this context, the discovery of previously undescribed taxa from human keratinized tissues is of particular interest, as it may improve our understanding of fungal diversity associated with the skin and of the traits that facilitate adaptation to this ecological niche.

Here, we describe such a novel hyphomycete isolated from eczematous plantar human stratum corneum. Phylogenetic analyses placed the isolate close to *Pseudogeomyces* but without close relatedness to any hitherto described species and genus. Therefore, the fungus is described in the manuscript as *Dermkielia cavawata* gen. nov. et sp. nov., IF 905164.

## 2 Materials and Methods

### 2.1 Clinical Procedure

**FIGURE 1:**
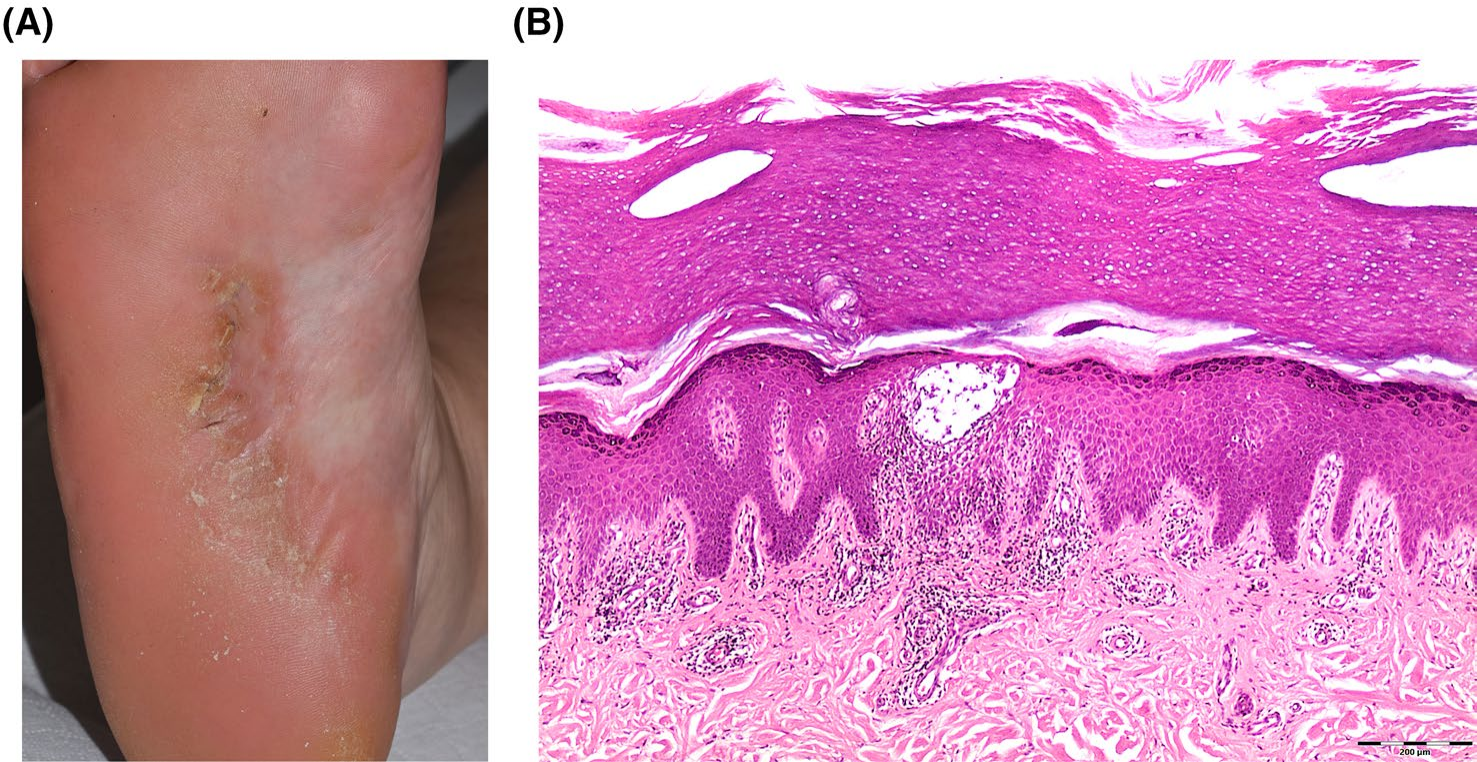
Skin lesion. (a) Ill‐defined erythematous, exfoliative lesion on right planta pedis, corresponding to subacute eczema; source of the described fungus. (b) Histopathology of the plantar lesion, haematoxylin‐eosin‐staining. Hyper‐ and parakeratosis, acanthosis, spongiosis, intraepidermal vesicle and mononuclear infiltrates indicate eczema.

A 64‐year‐old woman presented in our outpatient clinic with hyperkeratotic scaly and erythematous lesions on the soles of her feet (Figure 1). Her medical record and recent medical history were reviewed, and a dermatological clinical examination was done. In this process, lesional scales were collected for conventional mycological diagnostics (see below). Furthermore, a plantar biopsy was taken for histopathology.

### 2.2 Phenotypic Mycological Assessments

All assays and physiological tests were performed at least in duplicate, following generally accepted procedures established for hyphomycetes, including dermatophytes [10, 11, 12, 13, 14, 15, 16, 17].

Cultures were grown at 26 (± 1) °C on dermatophytes agar containing 0.4 g/L cycloheximide and 0.1 g/L chloramphenicol (DA; Sifin, Berlin, Germany). Four‐millimetre plugs of fresh colonies cut from these plates were used for inoculation of the following growth media (all incubated at 26°C ± 1°C): DA, Sabouraud agar supplemented with gentamicin and chloramphenicol (SGC2; BioMèrieux, Marcy‐l'Etoile, France), potato‐dextrose agar (PDA, Sigma‐Aldrich Chemie GmbH, Schnelldorf, Germany), Takashio agar (TA) and Czapek‐yeast‐agar (CYA; Himedia Laboratories Pvt. Ltd., Maharashtra, India). Additional cultures on DA, SGC2 and PDA were incubated at 15°C ± 1°C. All cultures were examined after 2, 3 and 4 weeks for distinctive morphological features and assessed microscopically for significant structures using transparent adhesive tape preparations stained with lactophenol blue. Ascomata production was checked after 12 weeks on DA, SGC2 and PDA. Slide cultures for additional microscopic assessments were prepared on DA, SGC2, PDA and CYA at 26°C and assessed after 2–4 weeks.

Thermotolerance was tested at 37 (± 1) °C on DA, SGC2 and CYA, and growth at 5°C was assessed on the same media. The following physiologic tests were done at 26 (± 1) °C: urease test on urease agar slants (Becton Dickinson BBL, Sparks, MD, USA), with readings after 7 and 10 days; salt tolerance on SGC2 supplemented with 1%, 2%, 4% and 6% NaCl; vitamin dependency on *Trichophyton* agar 1 (TA1; Becton Dickinson Difco, Sparks, MD, USA); growth on human hair and hair perforation according to Ajello [18]; growth on human stratum corneum as described previously [9]; growth on hen's feathers and garden soil as described previously [10]; utilization of cellulose and inulin on agar media prepared with these substrates [10]; lipolytic activity on Tween 80 opacity medium [19]. Bromocresol purple casein agar (BPCA) and blood‐agar (BA); Mueller Hinton II Agar with 5% horse blood (Mast Diagnostika, Reinfeld, Germany) were used to screen for haemolysis, proteolysis and alteration of pH, respectively [12]. Susceptibility to itraconazole and terbinafine was evaluated using an agar‐based screening method for dermatophytes on RPMI agar plates [10, 20].

### 2.3 DNA Amplification and Sequencing

A PCR screening test for dermatophytes (EUROArray Dermatomycosis) performed with culture material according to manufacturer's instructions was negative. Therefore, molecular identification was independently performed by the Institute of Microbiology (Charité, Berlin, Germany) and the Leibniz Institute DSMZ‐German Collection of Microorganisms and Cell Cultures (Braunschweig, Germany), as described previously [10, 15]. At the Institute of Microbiology (Charité), PCR amplification of the internal transcribed spacer (ITS) region (ITS1, 5.8S rRNA gene and ITS2) was performed using primers V9D and LSU266 with the Phire Plant Direct PCR Master Mix (Thermo Scientific) without prior DNA extraction [21]. The PCR product was sequenced using primer ITS5 at LGC Genomics (Berlin, Germany) [22]. Independently, at the DSMZ, DNA was extracted using the Epicentre MasterPure Yeast DNA Purification Kit according to the manufacturer's instructions. The ITS region and the D1/D2 domains of the LSU rRNA gene, was amplified using primers ITS1F and LR5 [23, 24]. The PCR product was purified with QIAquick PCR Purification Kit (Qiagen, Germany) and sequenced with ITS4 and NL4 primers [25]. Chromatograms were assembled, checked and corrected with Sequencher 5.4.6 (Gene Codes Corp., USA). Nucleotide ITS and LSU sequences of the new isolate were submitted separately to BLASTn searches against the NCBI database (www.ncbi.nlm.nih.gov/BLAST), using both the complete and RefSeq databases, to determine the most closely related fungal species.

### 2.4 Alignment and Tree Construction

Based on the results of pair‐wise sequence similarity results obtained from BLASTn searches against the NCBI GenBank database, sequences of type and reference material representing species of the family *Thelebolaceae* (*Thelebolales*, *Leotiomycetes*) were selected following the phylogenies in Zhang et al. [26, 27] Multiple sequence alignments were performed with the genomic sequences using the online version of the MAFFT algorithm (version 7) using the Q‐INS‐i strategy and allowing for gappy regions [28]. The final dataset comprised 31 taxa representing the genera *Pseudogymnoascus*, *Geomyces*, *Solomyces*, *Gymnostellatospora* and *Pseudogeomyces*. *Leuconeurospora pulcherrima*, *Pseudeurotium ovale* and *Pseudeurotium zonatum* were used as outgroup taxa. The concatenated ITS and LSU alignment consisted of 1400 positions, including 581 bp from ITS and 819 from LSU. This combined dataset was used to infer a combined ITS‐LSU phylogenetic tree. Phylogenetic relationships were inferred by the maximum likelihood (ML) method under the general time reversible (GTR) model, as implemented in RaxML GUI version 2.0.16. The GTR + G + I substitution model was applied, and node support was assessed with 1000 thorough bootstrap replicates [29].

## 3 Results

### 3.1 History and Clinical Assessment

During the preceding year, the patient had been diagnosed elsewhere with toenail onychomycosis caused by *Trichophyton rubrum*. For its treatment, several topical and systemic antifungal agents had subsequently been prescribed in a medical practice. In addition, nail material had intermittently been submitted to our mycological laboratory for diagnostic evaluation. All these tests consistently yielded negative KOH mounts and negative culture results. However, PCR‐based screening (EUROArray Dermatomycosis) occasionally detected *Meyerozyma guilliermondii*, *Scopulariopsis brevicaulis* (at low copy numbers) and *Neocosmospora* (*Fusarium*) *solani*. When the patient presented to our clinic, her plantar skin lesions (Figure 1a) were clinically diagnosed as eczema. In addition, dystrophic toenails and fingernails were observed, and this nail disorder was diagnosed as ungual lichen planus. The previous intermittent PCR findings were interpreted as transient fungal colonization. Nevertheless, due to the patient's complex history, differing prior diagnoses and earlier mycological findings, both plantar tinea and plantar lichen planus required definitive exclusion. For this purpose, a biopsy was taken from the plaque on her left sole. Histopathology revealed characteristics typical of subacute eczema (Figure 1b), and no fungal elements were detected by PAS staining. Lesional plantar scales were also examined for fungal presence. No fungi were observed in KOH mounts, whereas PCR (EUROArray Dermatomycosis) detected *Fusarium solani* and a hyphomycete was isolated at 26°C on DA with cycloheximide. This fungus was not identified as a skin pathogen and was therefore considered clinically irrelevant. Consequently, our clinical diagnosis of plantar eczema, supported by histopathological findings, was retained. Treatment with acitretin was recommended, but, unfortunately, the patient was lost for follow‐up. For completeness, and out of academic interest, the cultured hyphomycete was subjected to a comprehensive mycological characterization in our laboratory (see below).

### 3.2 Phenotype

**FIGURE 2:**
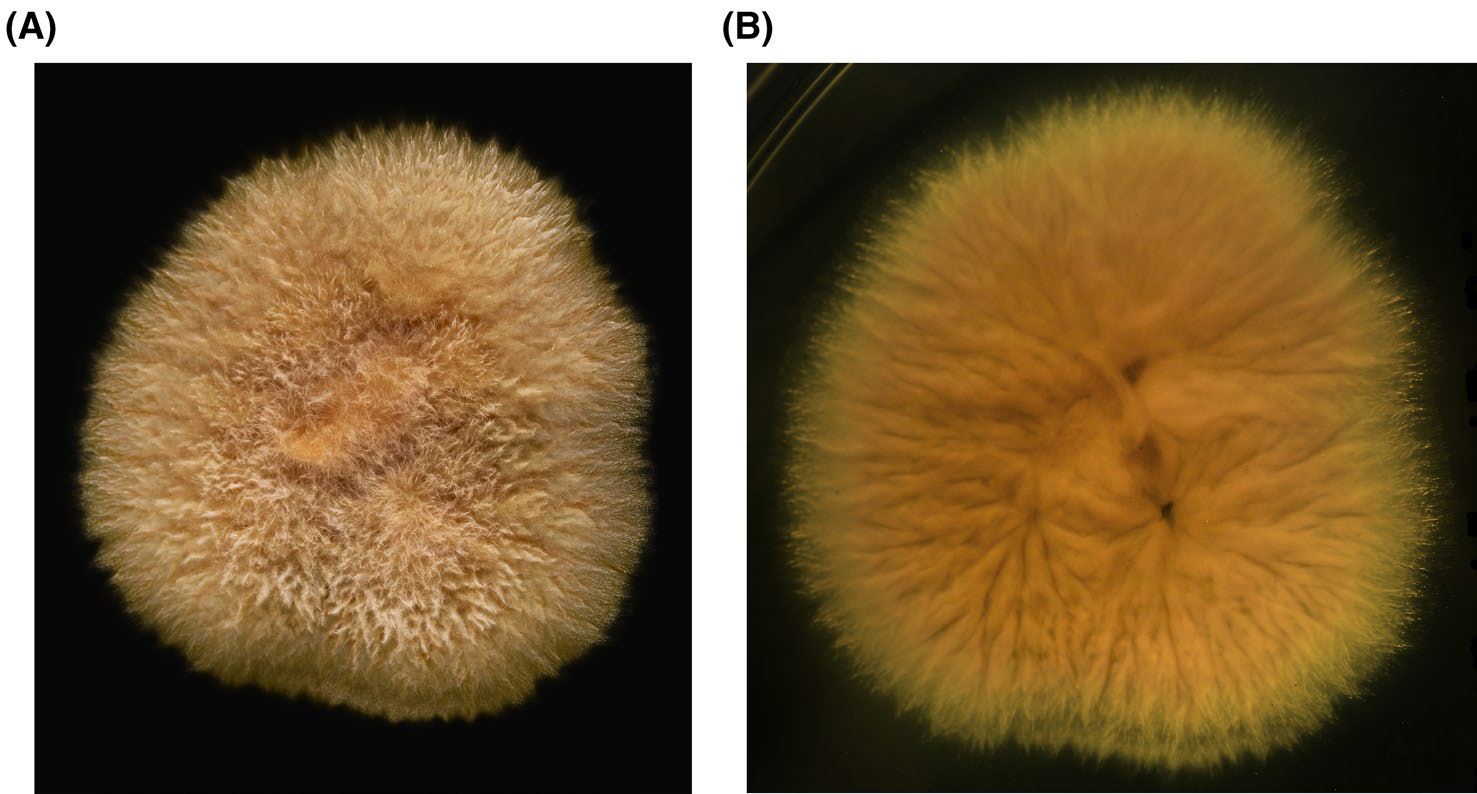
Culture of *Dermkielia cavawata* on dermatophytes agar. (a) Culture obverse, 26°C, 2 weeks. (b) Culture reverse, 26°C, 2 weeks.

The isolate grew well at 26°C on DA, SGC2, PDA and CYA, reaching colony diameters of approximately 19 mm, 25 mm, 30 mm and 20 mm, respectively, after 2 weeks of incubation. Colony morphology was similar across all these media. The cultures produced flat colonies with beige obverse, sparse aerial mycelium with coarse hanks and shallow irregular radial furrows (Figure 2a). Margins were flat and slightly fringed. The reverse side displayed somewhat more intense yellowish‐beige pigmentation (Figure 2b). At 15°C, growth on DA, SGC2 and PDA was reduced, with colony diameters of approximately 10, 15 and 15 mm, respectively, after 2 weeks of incubation. However, colony morphology remained highly like that observed at 26°C.

**FIGURE 3:**
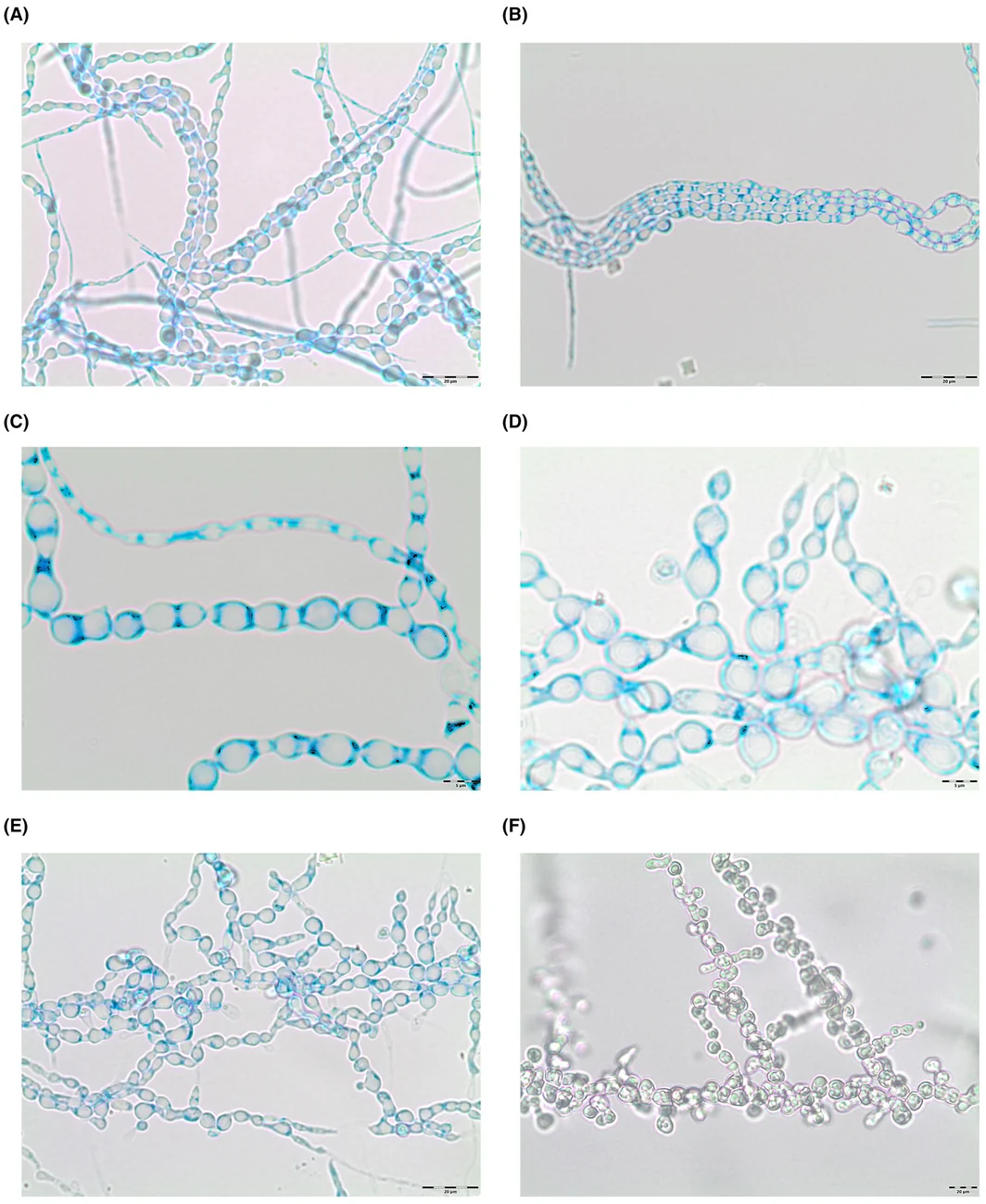
Microscopic features of *Dermkielia cavawata* in microcultures. (a) Hyaline, thin‐ and smooth‐walled septate hyphae and thicker hyphae with a tendency to form synnemata; densely arranged intercalary round spores. Lactophenol blue stain, 40‐fold. (b) Bundle of hyphae with intercalary conidia. Lactophenol blue stain, 40‐fold. (c) Intercalary conidia with diameters up to 5 μm. Lactophenol blue stain, 100‐fold. (d) Spores and initial chlamydospores. Lactophenol blue stain, 100‐fold. (e) Hyphal network with spores aligned similar to strings of beads. Lactophenol blue stain, 40‐fold. (f) Submersed hyphae in old culture, transformed into chains of spores and chlamydospores with diameters up to 10 μm. 40‐fold.

Microscopically, colonies on DA, SGC2 and on CYA consisted of hyaline, thin‐ and smooth‐walled branched and septate lean hyphae that thickened in the course of time and tended to form synnemata (Figure 3a,b). Intercalary, densely arranged, round to subglobose spores developed within the hyphae (Figure 3a–c). These spores were arranged like strings of beads, either contiguous or separated by short hyphal segments (Figure 3b). Spore diameters increased progressively, reaching more than 5 μm (Figure 3c,d). In older cultures, dense networks of such strings with an appearance like bead chains were prominent (Figure 3e). Submersed hyphae in old cultures were completely transformed into chains of spores, with some spores reaching diameters of 10 μm, consistent with chlamydospore morphology (Figure 3f). Neither microconidia nor macroconidia were observed. Ascomata did not develop on any of the employed media under the conditions tested.

**FIGURE 4:**
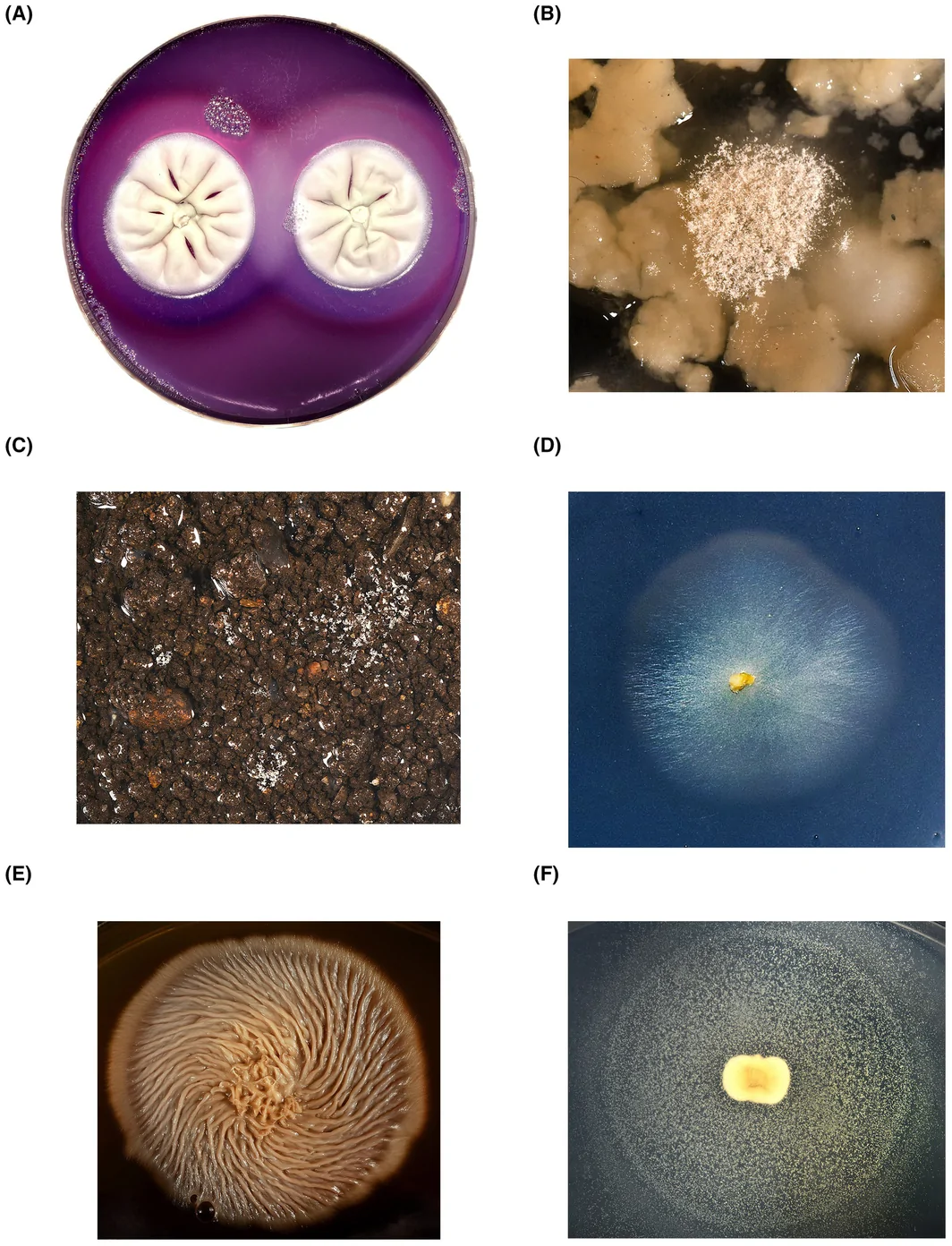
Physiologic characteristics of *Dermkielia cavawata*. (a) Culture on bromocresol purple casein agar, 26°C, 4 weeks. Pleated and greenish colony obverse surrounded by a clear halo indicating proteolysis. (b) Culture on stratum corneum with sparse white mycelium, 4 weeks, 26°C. (c) Culture on garden soil with sparse white mycelium, 4 weeks, 26°C. (d) Culture on inulin agar, 3 weeks, 26°C. Thin, transparent colony, approximately 20–25 mm in diameter. (e) Culture on Sabouraud agar supplemented with 4% NaCl, 4 weeks, 26°C. Conspicuous rope‐like structures in a spiral pattern on the obverse; dark violet pigmentation of reverse. (f) Culture on Tween 80 opacity medium, 2 weeks, 26°C. Precipitation indicates lipolysis.

No growth was observed at 37°C on DA, SGC2 and CYA. At 5°C, growth was slow but clearly positive, resulting in colony diameters on DA, SGC2 and CYA of 10 mm, 12 mm and 8 mm, respectively, after 8 weeks of incubation. The following physiological characteristics were obtained at 26°C. On BA, the thallus reached only a few millimetres in diameter after 3 weeks and was surrounded by an approximately 2 mm wide clear zone indicating β‐haemolysis. On BPCA, colonies were domed with off‐white obverse, velvety and strongly folded, attaining a diameter of approximately 35 mm after 4 weeks and surrounded by a clear halo indicating proteolytic activity (Figure 4a). Good growth on vitamin free TA1 indicated independence from exogeneous vitamins. The urease test was positive after 7 days. The isolate exhibited slow growth on human stratum corneum (Figure 4b) and on human hair, where it caused corrosion of the hair shafts without perforation. No growth was observed on hen's feathers, whereas slow growth occurred on garden soil (Figure 4c). On media containing cellulose, inulin, or both, thin transparent colonies developed, reaching diameters of 20–25 mm after 3 weeks (Figure 4d). Increasing NaCl concentrations (0%, 1%, 2%, 4%, 6%) in SGC2 progressively reduced the thallus diameters at both 26°C and at 15°C, from approximately 25 to 8 mm and from 15 to 4 mm, respectively. An increase of NaCl concentration also stimulated a dark violet pigmentation of the culture reverse and peculiar rope‐like structures in a spiral pattern on the culture surface, most prominent with 4% NaCl (Figure 4e). On Tween 80 opacity medium a precipitation of crystals indicated lipolysis (Figure 4f). On control RPMI plates, the thallus reached diameters of approximately 18 mm after 2 weeks. In the presence of 0.125 mg/L terbinafine, growth was reduced to approximately 10 mm, whereas no inhibition was observed at 0.016 mg/L terbinafine. In contrast, itraconazole at 1.0 mg/L completely inhibited growth.

### 3.3 Molecular Characteristics

**FIGURE 5:**
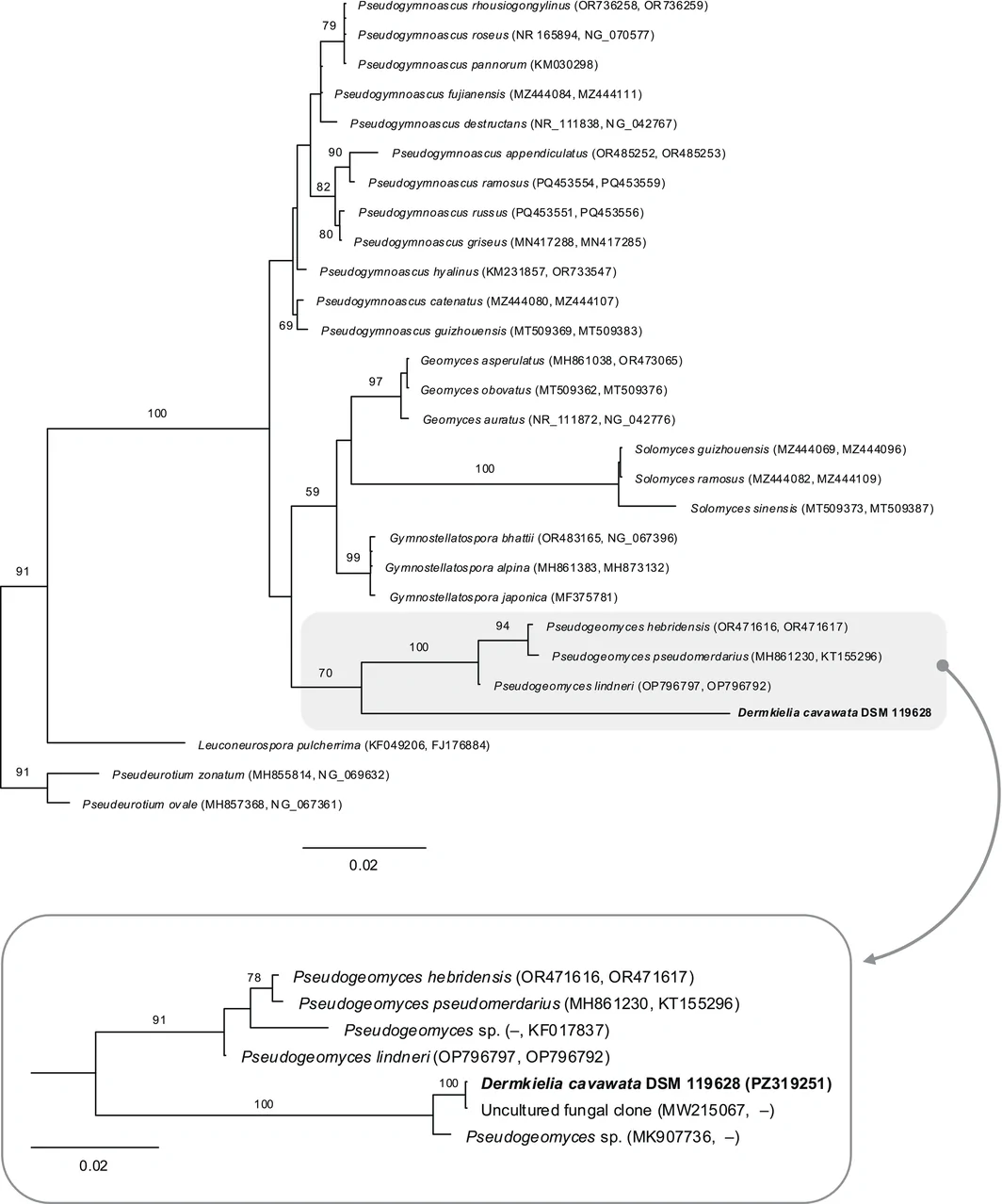
Combined ITS‐LSU phylogenetic maximum‐likelihood tree. Maximum likelihood analysis of concatenated sequences of the internal transcribed spacer (ITS) regions and D1/D2 domains of the LSU rRNA gene showing the placement of *Dermkielia cavawata* DSM 119828 (GenBank PZ319251) relative to the genera *Pseudogeomyces*, *Pseudogymnoascus*, *Geomyces*, *Solomyces* and *Gymnostellatospora*. Numbers given on branches are frequencies (> 60%) with which a given branch appeared in 1000 bootstrap replications. GenBank accession numbers used for the construction of the phylogenetic tree are provided in parentheses. The tree is rooted with the species *Pseudeurotium ovale* and *Pseudeurotium zonatum* .

Nucleotide sequences of the isolate (NCBI GenBank accession PZ319251) showed relatively low similarity in BLASTn searches against the NCBI GenBank database, indicating a distant relationship to currently described species of the genus *Pseudogymnoascus* and other members of the family *Thelebolaceae* (*Leotiomycetes*, *Thelebolales*). The closest LSU matches were obtained with species of *Pseudogymnoascus* ( *P. hyalinus* , *P. pannorum*, *P. rhousiogongylinus*, *P. roseus* ) and *Gymnostellatospora* ( *G. alpina* , *G. bhattii*, *G. canadensis* , *G. frigida*), showing sequence similarities in the range of 98.0%–98.5%. In contrast, ITS region comparisons yielded lower similarity values of approximately 90% to species of *Gymnostellatospora*. Phylogenetic analysis included representative species of the genera *Pseudogymnoascus*, *Geomyces*, *Solomyces*, *Gymnostellatospora* and *Pseudogeomyces*, following previous phylogenetic analyses performed by Minnis and Lindner and Zhang et al. [30, 31] *Leuconeurospora pulcherrima*, *Pseudeurotium ovale* and *Pseudeurotium zonatum* were used as outgroup taxa. All included genera were resolved in the ML analysis with good statistical support (> 80%) (Figure 5). The isolate described here, *Dermkielia cavawata* (DSM 119628; Figure 5), formed a distinct lineage, branching basally to the genus *Pseudogeomyces* (clade O sensu Zhang et al.) [27] and was separated from other species by a considerable phylogenetic distance. The resulting topology was consistent with previous studies [27, 30, 31]. Although support for placement within *Pseudogeomyces* was moderate (ML bootstrap: 70%), the observed phylogenetic distance was comparable to that separating the genera *Geomyces* and *Solomyces* (Figure 5). This level of divergence suggests that the isolate should not be assigned to *Pseudogeomyces* but instead represents a distinct lineage warranting recognition at the genus level consistently with previous studies [26, 27, 30]. Accordingly, the new genus *Dermkielia* is introduced here to accommodate the novel species *Dermkielia cavawata* (see Taxonomy below). A comprehensive search of NCBI GenBank identified two additional ITS sequences (MW215067, MK907736) likely belonging to this lineage, as indicated by the phylogenetic analysis (with missing LSU data coded accordingly). This finding indicates that further species of this previously unrecognized and phylogenetically distinct genus may be discovered in the future.

## 4 Discussion

Our accidental discovery nicely illustrates that human skin affected by an inflammatory epidermal disorder may harbour unexpected fungi [9, 10, 15, 16, 32, 33, 34]. It further emphasizes that mycological findings must always be interpreted in their clinical context. In the present case, heterogeneous prior findings had led to repeated and likely unnecessary antimycotic treatments. In such situations, the isolation of a hyphomycete can easily result in a misdiagnosis of tinea. However, in our patient, a fungal infection was clearly excluded based on histology and because the isolate was not a known pathogen. Although the isolate was therefore considered clinically irrelevant, it was retained for further investigation.

The isolate formed whitish colonies on dermatophyte agar containing 0.4 g/L cycloheximide, and microscopy revealed intercalary chains of spores, initially suggesting affinity with *Arthrographis* species. However, the predominantly round spores did not correspond to typical arthrospores, and additional culture conditions failed to induce the formation of conidiophores or other diagnostic structures. The metabolic and physiologic properties of the isolate, such as growth at 5°C, persistence in soil, utilization of protein substrates like hair keratin, and the ability to metabolize cellulose and inulin, combined with salt tolerance, suggested a geophilic nature of the fungus. These characteristics are reminiscent of members of the *Onygenales*, *Geomyces* and *Pseudogymnoascus* [5, 17, 31, 35], which are known to include opportunistic geophilic dermatophytes capable of colonizing human skin under suitable conditions. This prompted molecular identification of the isolate.

Phylogenetic analysis robustly placed the novel species *Dermkielia cavawata* within the family *Thelebolaceae* (*Leotiomycetes*, *Thelebolales*), in a clade comprising the genera *Pseudogymnoascus*, *Geomyces*, *Solomyces*, *Gymnostellatospora* and *Pseudogeomyces*. More specifically, the species was positioned in proximity to *Pseudogeomyces*. However, the high level of nucleotide sequence divergence and the substantial phylogenetic distances observed indicate that the isolate should not be assigned to *Pseudogeomyces*, but instead represents a distinct lineage, consistent with previous studies [26, 31]. The limited number of related sequences available in GenBank provides initial insights into the ecology of this lineage. A potential soil‐borne origin of *D. cavawata* is supported by DNA metabarcoding data from soils in Lithuania [36]. In addition, a closely related sequence, *Ascomycota* sp. isolate Z112 (ITS: MK907736.1) that shows 98% similarity, was obtained from *Betula pendula* shoot dieback in Latvia, supporting an environmental source of the species. Further searches for related environmental sequences using the GlobalFungi platform identified ITS sequences from soil samples showing approximately 90% similarity to *D. cavawata* (data not shown). These findings also support a predominantly soil‐associated origin of this phylogenetic lineage.

The phylogenetic placement (Figure 5) also showed that *D. cavawata* is distant from the *Onygenales*, an order that includes many keratinolytic and human pathogenic species [8], but is closely related to the geophilic species *Pseudogeomyces lindneri*. The genus *Pseudogeomyces*, established only recently in 2023 with *Pseudogeomyces lindneri* as type species [27], belongs to the family *Thelebolaceae*, order *Thelebolales*, class *Leotiomycetes*, a monophyletic class within the *Ascomycota* phylum. *Pseudogeomyces lindneri* detected in soils from China and the USA produces intercalary conidia, like those observed in our isolate. However, *Pseudogeomyces lindneri* also forms conidiophores and hyaline rough conidia [27], features lacking in our isolate. In contrast, closely related genera such as *Solomyces*, *Geomyces* and *Pseudogymnoascus* are characterized by distinct forms of conidiogenesis, all of which result in the production of microconidia [26, 27, 31, 35], while *Gymnostellatospora* produces ascomata [37]. The absence of conidiophores, microconidia or ascomata in *Dermkielia cavawata*, combined with its distant phylogenetic position, supports its classification in a new genus, here designated *Dermkielia*, named after the dermato‐mycological laboratory in Kiel.

*Dermkielia cavawata* was isolated from human eczematous plantar skin and was interpreted as an accidental superficial contamination of the stratum corneum, possibly acquired through barefoot exposure. Nevertheless, its keratinolytic, proteolytic, haemolytic and lipolytic properties suggest that, under suitable conditions, it might colonize or even infect epidermis. The species may have a reduced susceptibility to terbinafine, which may become clinically relevant should pathogenicity be demonstrated in the future.

Identification of *D. cavawata* by conventional mycological methods is challenging. Due to its resistance to cycloheximide, this fungus grows well on culture media designed to selectively isolate dermatophytes and may, therefore, be mistaken for them in busy routine diagnostics. Macroscopic colony morphology on common mycological agars is not distinctive. However, the peculiar spiral pattern of rope‐like structures on NaCl‐supplemented Sabouraud agar (Figure 4e) might come in useful as a discriminatory morphologic feature once comparison data of related species are available. Microscopically useful clues in older cultures on common agars are networks of bead chain‐like connected spores combined with the absence of micro‐ and macroconidia. Physiological tests distinguishing *D. cavawata* from *Pseudogeomyces* are not available at the time of the investigation, because comparable data are lacking in the description of *Pseudogeomyces lindneri*. Currently, an unambiguous identification of *D. cavawata* requires ribosomal RNA sequencing.

In recent years, the number of keratinophilic fungi identified in soils has increased, and their phylogenetic relationships are gradually unveiled by molecular phylogenetic approaches [26, 31, 35, 38]. The description of *D. cavawata* contributes to this expanding research field. Further studies are needed to clarify the ecology, physiology, and potential clinical relevance of such fungi. Given their keratinophilic properties, they should be monitored as potential agents of tinea. Their biology and phylogenetic placement should be considered carefully to ensure accurate taxonomic classification [8, 39, 40].

## 5 Taxonomy

*Dermkielia*, J. Brasch, Y. Gräser, K. Voss, K. A. Langen & A. Yurkov, gen. nov.

Index Fungorum: IF 905143.

Etymology: The generic name refers to the dermato‐mycological laboratory of the Department of Dermatology at the University Hospitals of Schleswig‐Holstein (UKSH), Campus Kiel, in Kiel, Germany, in recognition of its contribution to medical mycology.

Diagnosis: Differs from phylogenetically related genera *Pseudogeomyces*, *Pseudogymnoascus*, *Geomyces*, *Solomyces* and *Gymnostellatospora* by the absence of conidiophores, microconidia and ascomata, and by the formation of intercalary, globose to subglobose spores aligned like bead chains that frequently develop into chlamydospores.

Description: *Dermkielia* is classified in the family *Thelebolaceae*, order *Thelebolales*, class *Leotiomycetes*, phylum *Ascomycota*. The genus is separated based on its phylogenetic placement in the combined ITS‐LSU maximum‐likelihood tree. Colonies are flat, beige, furrowed, with sparse aerial mycelium. Intercalary globose to subglobose spores are formed, often in strings like bead chains; these may develop into chlamydospores up to 10 μm in diameter. Conidiophores and ascomata were not observed.

Type species: *Dermkielia cavawata* Brasch et al. (IF 905164).

Note: This genus is currently monotypic and established to accommodate the type species *Dermkielia cavawata* (see below). A potential additional member of the genus is represented by the ITS sequence labelled as *Ascomycota* sp. isolate Z112 (NCBI GenBank ITS: MK907736.1), obtained from birch ( *Betula pendula* ) shoot dieback in Latvia.

*Dermkielia cavawata*, J. Brasch, Y. Gräser, K. Voss, K. A., Weidinger S. & A. Yurkov, sp. nov.

Index Fungorum: IF 905164.

Etymology: The species name is a vocalized acronym derived from the submitting author's children in appreciation of their mycotolerance.

Holotype: Germany, Schleswig‐Holstein, Kiel, isolated from human skin, 28 June 2024, coll. J. Brasch; original isolate designation Kiel 1332–2024, preserved in a metabolically inactive state in the German Collection of Microorganisms and Cell Cultures, Braunschweig, Germany as DSM 119628 (holotype); metabolically inactive isotypes CBS 154290 in the CBS collection of the Westerdijk Fungal Biodiversity Institute, Utrecht, The Netherlands and IHEM 29324 in the BCCM/IHEM Fungal Collection: Human & Animal Health, Brussels, Belgium.

Molecular characteristics (holotype): GenBank/ENA/DDBJ PZ319251, ribosomal ITS and large subunit ribosomal RNA gene, partial sequence.

Description: Good growth at 26°C on conventional mycological agars. On Sabouraud glucose agar and dermatophyte agar, cultures with flat beige obverse, sparse aerial mycelium with coarse hyphal aggregates, shallow irregular radial furrows and slightly fringed flat margins. Reverse with slightly stronger yellowish‐beige pigmentation. Growth on human stratum corneum, human hair and garden soil. Hyphae hyaline, thin‐ to smooth‐walled, branched and septate, thickening over time. Intercalary, densely arranged, round to subglobose spores develop within the hyphae and are arranged similar to bead chains, either contiguous or separated by short hyphal remnants. Spores partly develop into chlamydospores up to approximately 10 μm in diameter. In older cultures, hyphae may be nearly completely transformed into chains of spores. No microconidia or macroconidia detected. Sexual reproduction not observed. Keratinolytic, proteolytic, cellulolytic, haemolytic, lipolytic and urease positive. Growth in the presence of up to 6% NaCl and 0.4 g/L cycloheximide. No vitamin supplementation required. No growth at 37°C; slow growth at 5°C. Susceptible to itraconazole and less susceptible to terbinafine.

Habitat and ecology: *Dermkielia cavawata* is presumably a geophilic species, possibly linked to forest ecosystems and plant rhizosphere, although initially isolated from human skin.

Distribution: Currently known from the type locality in Germany. Related environmental ITS sequences from Lithuania [Marciulyniene 2021] suggest a broader distribution in forest habitats.

Material examined: 1332‐2024 (= DSM 119628 = CBS 154290 = IHEM 29324), from human skin, Kiel, Schleswig‐Holstein, Germany.

## Author Contributions

**Yvonne Gräser:** investigation, writing – review and editing, methodology. **Jochen Brasch:** conceptualization, investigation, methodology, writing – review and editing, project administration, supervision. **Andrey Yurkov:** investigation, writing – original draft, writing – review and editing, software, methodology. **Karen Voss:** investigation. **Stephan Weidinger:** writing – review and editing.

## Conflicts of Interest

The authors declare no conflicts of interest.

## Data Availability

The data that support the findings of this study are available from the corresponding author upon reasonable request.
